# Electroencephalography Signatures for Hepatic Encephalopathy in Cirrhosis Patients Treated with Proton Pump Inhibitors: An Exploratory Pilot Study

**DOI:** 10.3390/biomedicines10123040

**Published:** 2022-11-24

**Authors:** Pan Zhang, Lizhi Zhou, Li Chen, Zhen Zhang, Rui Han, Gangwen Guo, Haocheng Zhou

**Affiliations:** 1 Department of Infectious Diseases, Third Xiangya Hospital, Central South University, Changsha 410013, China; 2 Department of Infectious Diseases, Xiangtan Central Hospital, Xiangtan 411100, China; 3 Department of Pain, Third Xiangya Hospital and Institute of Pain Medicine, Central South University, Changsha 410013, China; 4 Hunan Key Laboratory of Brain Homeostasis, Central South University, Changsha 410013, China

**Keywords:** hepatic encephalopathy, cirrhosis, proton pump inhibitor, risk factor, EEG, cortical, neural oscillation

## Abstract

(1) Background: Hepatic encephalopathy (HE) is a common complication in cirrhosis patients, and recently, clinical evidence indicates that a higher risk of HE is associated with the usage of proton pump inhibitors. However, the cortical mechanism underlying this neurological disorder of HE remains unknown. (2) Methods: We review the medical recordings of 260 patients diagnosed with liver cirrhosis between January 2021 and March 2022 in one tertiary hospital. Logistic regression analyses were performed to identify the risk factor of HE development. To examine the relationship between cortical dynamics and the administration of proton pump inhibitors, resting-state electroencephalograms (EEGs) were conducted in cirrhosis patients who were treated with proton pump inhibitors. (3) Results: About 28.5% (74 out of 260) of participants developed secondary HE in this study. The logistics regression model indicated that multiple risk factors were associated with the incidence of secondary HE, including proton pump inhibitors usage, white blood cell and neutrophil counts, hemoglobin, prothrombin time activity, and blood urea nitrogen. A total of twelve cirrhosis patients who were scheduled to use proton pump inhibitors consented to performing electroencephalogram recordings upon admission, and eight of twelve participants were diagnosed with HE. Spectral analysis revealed that the decrease in alpha oscillation activities was potentially associated with the development of HE. (4) Conclusions: Our data support the susceptibility of secondary HE in cirrhosis patients treated by proton pump inhibitors. One potential cortical mechanism underlying the neurological disease is the suppression of alpha oscillations in the brain.

## 1. Introduction

It has been estimated that the aged-standardized prevalence of cirrhosis was about 0.7% in China, with the leading cause being the hepatitis B virus [1]. Multiple complications of cirrhosis significantly reduced the quality of life and accounted for the increasing mortality, including ascites, varices, hepatocellular carcinoma, hepatic encephalopathy (HE), hepatopulmonary syndrome, and coagulation disorders [2]. The featured neurological dysfunction was the most devastating complication of cirrhosis; namely, the HE is frequently reported to be associated with the medical usage of benzodiazepines, opiates, and proton pump inhibitors (PPIs) [3]. PPIs remains one of the most commonly used medication in cirrhosis populations despite its abuse in clinical practice [4,5]. Emerging evidence suggested the potential link between PPI applications and HE development [4,6,7,8]. Thus, it is essential to identify the susceptibility of HE in patients with advanced liver disease during PPI treatments.

Functional magnetic resonance imaging may provide morphological evidence as one potential bio-marker for exploring the neural correlates of cognitive deficits [9]. However, the great cost and complicated data processing may hinder clinical applications. Alternatively, the electroencephalogram is relatively easy to perform for the diagnosis of the neurological disease, including epilepsy, autism spectrum disorder, and Alzheimer’s disease [10,11]. Furthermore, emerging evidence has supported the diagnostic value of quantitative EEGs in terms of survival and the risk of developing overt HE [12,13]. However, the relationship between the cortical dynamics and HE development during PPIs management remains uncertain. In this study, we initially reviewed the medical data of 260 cirrhosis patients to confirm the role of PPIs in the development of secondary HE in hospitalized patients. Next, we aimed to investigate the potential cortical signature of HE candidates who received PPI treatment.

## 2. Materials and Methods

### 2.1. Patients and Study Design

This study was conducted in accordance with the guidance of the Helsinki Declaration and approved by the Ethics Committee of The Third Xiangya Hospital, Central South University, China (NO. 222128). The study was registered at chictr.org.cn accessed on 29 June 2022 (ChiCTR2200061590).

**Figure 1:**
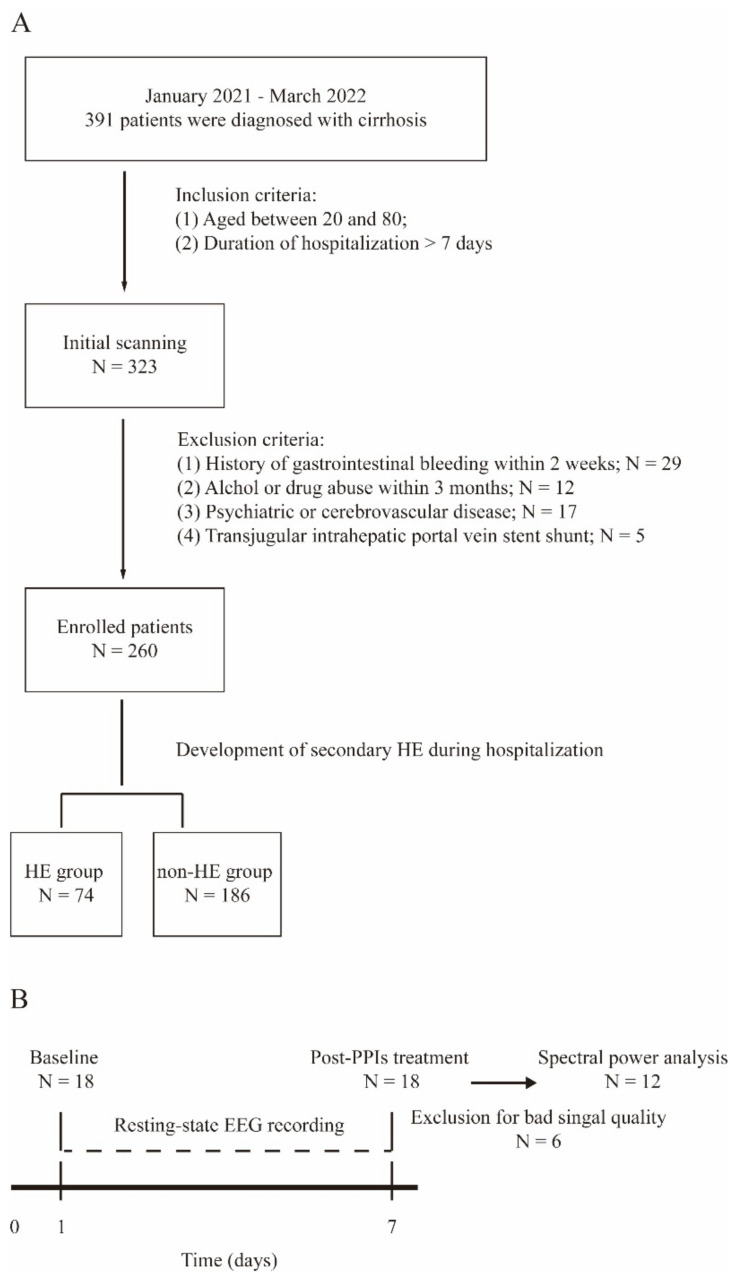
Study protocol for the Experiment One and Two. (**A**) Flow chart of patient selection for the first part of the study. (**B**) Schematic of EEG recording protocol for the second experiment.

In the first experiment, we retrospectively reviewed the medical recordings of 260 patients who were diagnosed with liver cirrhosis using ICD-9-CM codes at The Department of Infectious Diseases, Third Xiangya Hospital. The participants were then grouped into HE and non-HE sub-groups according to the development of secondary HE during hospitalization. HE is characterized by personality changes, intellectual impairment, and a depressed level of consciousness, which was classified into five degrees based on the severity of disease [14]. The initial search of HE incidences was performed by scanning the home page of electrical medical recording systems with the ICD-9-CM code (K72.903), which was followed by the manual verification of the medical records provided by one independent researcher (L.Z.). The classification of HE is provided in Table 1. We did not obtain information on disease severities for the first experiment due to the retrospective design of the study. However, we assessed the severity of HE in the prospective EEG study. The flow chart for patient selection and inclusion and exclusion criteria are shown in Figure 1A.

In the second experiment, eighteen cirrhosis patients who were scheduled to take PPI therapy during hospitalization were enrolled and consented to performing the EEG recording. The first session of EEG recordings was conducted upon admission, and the second session was accomplished one week after PPIs treatment. Six participants were excluded for poor quality EEG signals. The schematic of experiment two is shown in Figure 1B.

### 2.2. Resting-State EEG Recording

To evaluate the cortical features of patient with cirrhosis, resting-state EEG recordings were conducted as previously described [15]. The EEG signal was recorded in one quiet, temperature-controlled, and electrically shielded office. Participants were required to remain salient and awake during the EEG recording with eyes closed. One 16-channel bio-sensor (Cyton & Daisy, OpenBCI, Brooklyn, NY, USA) was used for the acquisition of data, which was connected to one electrode cap. The sampling rate of the EEG collection process was 128 Hz, and the impedance of each recording channel was kept below 10 KΩ to guarantee the quality of the EEG signal. The location of recording site and its region of interest is shown in Table 2. The Cz channel was selected as the reference electrode, and the Fpz channel was selected for the ground electrode.

### 2.3. EEG Signal Processing

The raw EEG data were restored by the OpenBCI Graphical User Interface and then extracted for further processing with the MATLAB 2021 software (R2018b, MathWorks, Natick, MA, USA). Offline EEG data preprocessing was accomplished by using the open-source EEGLAB toolbox [16]. One independent researcher (L.Z.) manually examined EEG raw traces to reject the artifacts and malfunctioning channels. Consequently, the continuous EEG data were filtered with one band-pass filter between 1 and 45 Hz and segmented into consecutive 2 s epochs. We also rejected the epochs with amplitudes over ±80 uV. Independent component analyses were then used to identify and rule out eye movement artifacts. This was followed by the collection of fifty artifact-free segments for the generation of datasets for quantitative analyses. To capture the cortical signature of potential HE patients, fast Fourier transforms were applied to calculate the spectrogram with the “spectopo.m” function script in EEGLAB. Five physiological sub-bands were then determined, including δ (delta, 0.5–4.0 Hz), θ (theta, 4.0–8.0 Hz), α (alpha, 8.0–13.0 Hz), β (beta, 13.0–30.0 Hz), and γ (gamma, 30.0–45.0 Hz).

### 2.4. Statistics

Data were presented with mean ± standard deviation, and the estimated risk was provided as odds ratios (ORs) with 95% confidence intervals (CIs). To assess the normality of data, Shapiro–Wilk tests were used. The chi-squared test or Fisher’s exact test were applied when comparing the categorical data, and Student’s t-test or Mann–Whitney U test were considered for continuous variables. To identify the independent risk factors of secondary HE, logistic regression analyses were performed using the variables, with a *p* value of less than 0.05 between the HE and non-HE groups. The spectral power density of each channel was calculated by averaging the data across epochs for each patient. Statistical analyses were conducted by the SPSS software (Version 26.0, Chicago, IL, USA). A *p* value of less than 0.05 was considered statistically significant.

## 3. Results

### 3.1. Comparison of Clinical Data between HE and Non-HE Cohort

About 28.5% (74 out of 260) of cirrhosis patients developed the secondary HE during hospitalization in this study. In the HE subgroup, almost eighty percent of participants were males, and 69.9% for the non-HE cohort. The white cell and neutrophil counts were significantly higher in the HE sub-group. The HE population presented generalized and worsened hepatic functions, as demonstrated by the lower platelet count and albumin and increased total bilirubin. In addition, significantly lower prothrombin activities and greater international normalized ratios may reveal the worsened dysfunction of coagulation in the HE cohort. The severity of liver diseases significantly increased in the HE cohort, as assessed by the Child–Pugh and MELD scores. Less PPIs (76.3%) were administrated in the non-HE sub-group, and 95.9% were administrated for the HE group. However, we did not observe significant differences in etiologies between two cohorts. The clinical data are provided in Table 3.

### 3.2. Risk Factor Associated with the Development of Secondary HE

In the univariate analysis, multiple variables were associated with the incidence of secondary HE in hospitalized patients, including white blood cell, hemoglobin, neutrophils, total bilirubin, albumin, blood urea nitrogen, potassium, prothrombin time activity, international standard ratio, MELD scoring, and PPIs application. This was followed by the multiple stepwise logistic regression analysis, which identified that white blood cell (OR = 1.972, 95%CIs 1.299–2.993), hemoglobin (OR = 0.978, 95%CIs 0.963–0.992), neutrophils (OR = 0.505, 95%CIs 0.317–0.805), prothrombin time activity (OR = 0.955, 95%CIs 0.936–0.975), blood urea nitrogen, (OR = 1.104, 95%CIs 1.018–1.198), and PPIs usage (OR = 7.867, 95%CIs 2.166–28.575) independently associated with the development of secondary HE (Table 4).

### 3.3. Comparison of EEG Patterns between HE and Non-HE Patients Treated with PPIs

**Figure 2:**
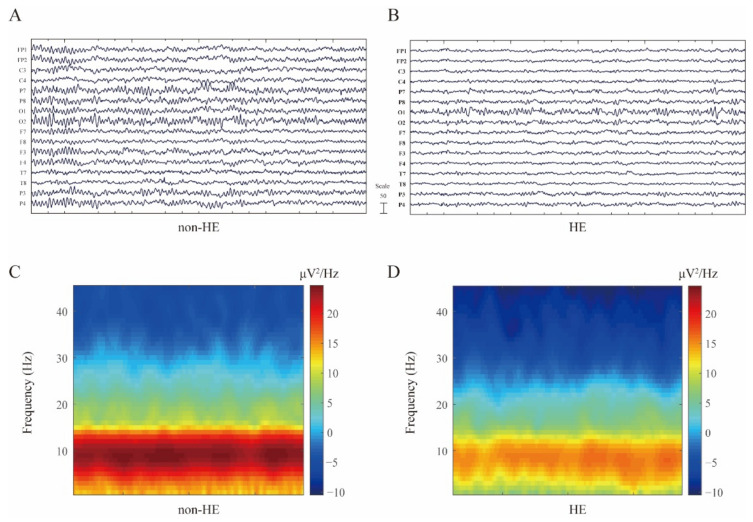
Comparison of EEG signal between non-HE and HE patients who received PPIs treatment. (**A**) Representative resting-state EEG traces in non-HE cohort, and (**B**) one with HE, respectively. (**C**,**D**) Comparison of spectrogram at distinct neurological condition (non-HE versus. HE).

A total of 18 cirrhosis patients who were scheduled to receive the standard PPI therapy (more than 1 week) consented to take the resting EEG recording. Six of them were excluded for further analyses for bad EEG signals. About 66.7% (8 out of 12) of these patients were diagnosed with HE after PPI treatments, and the baseline EEG data were recorded upon admission (Figure 2A,C). Infection may result in the development of HE, pneumonia was found in four HE cases and four with peritonitis, respectively. All the HE participants were classified into minimal HE according to the disease severity criteria (Table 1). In contrast, four patients who underwent baseline EEG recordings and PPI therapy (Figure 2B,D) did not present HE symptoms during hospitalization.

### 3.4. Alpha Oscillatory Activity Decreased in HE Candidates before PPI Therapy

**Figure 3:**
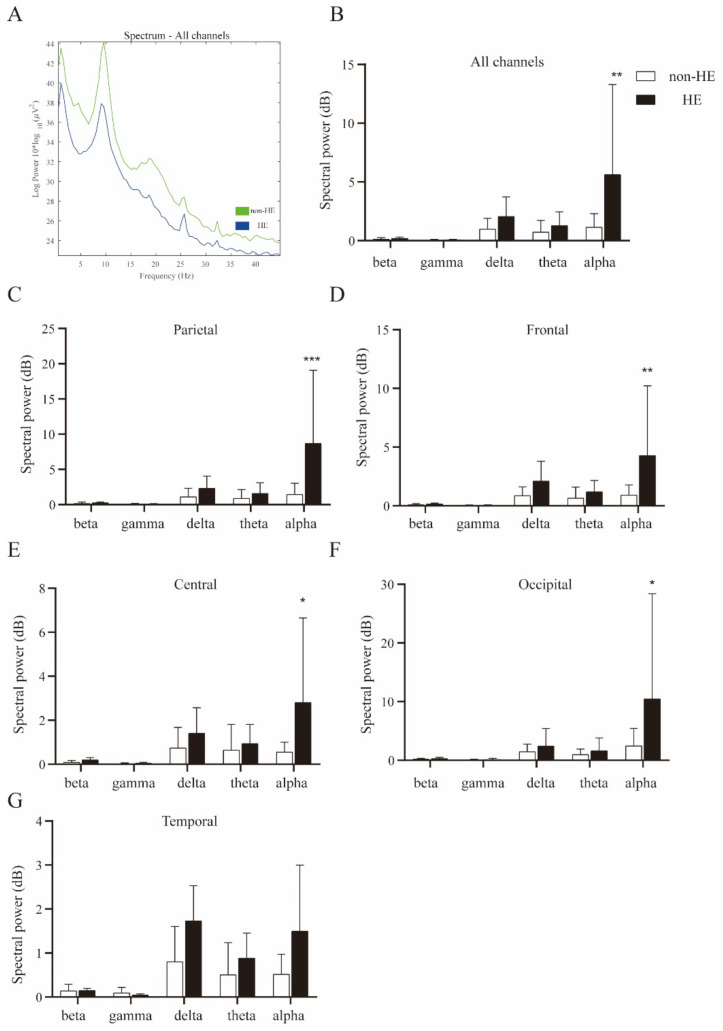
Reduction of alpha oscillations in the HE patients treated with PPIs. (**A**) Grand average spectral power was compared between non-HE and HE patients, (**B**) significant reduced alpha neural oscillation was associated with the HE development in cirrhosis patients under PPIs therapy. (**C**–**F**) Suppression of cortical alpha activity was detected in the parietal, frontal, central, and occipital, but NOT in the temporal region (**G**). A repeated-measures two-way ANOVA with post hoc Bonferroni tests. * *p* < 0.05, ** *p* < 0.01, *** *p* < 0.001.

Spectral analyses were then performed to capture the cortical signature of patients who received PPI treatments. The grand average spectral power significantly decreased in the HE sub-group at the sub-band of the alpha rhythm (Figure 3A,B). To detect the potential source of distinct cortical oscillations, we compared the spectral power of alpha activities at different brain regions, as shown in Table.2. Likely, only alpha oscillations were significantly enhanced across the frontal, central, parietal, and occipital sites (Figure 3C–F). No statistical significance with respect to spectral power densities was found in the temporal region (Figure 3G).

## 4. Discussion

Cirrhosis is one late-stage hepatic disease caused by multiple etiologies, including viral infection, fatty liver, autoimmune, chronic biliary, and cardiovascular disease [17]. Given its increasing prevalence in recent years [18], initiatives to prevent its progress and the severe comorbidity are needed, among which the HE remains one common yet debilitating complication in the cirrhosis population. Thus, the early recognition of HE is key to improving the clinical outcomes for patients with advanced liver disease, especially for those treated with multiple medications, resulting in potential liver or neurological damage. In this study, we focused on the risk factor of secondary HE in the hospitalized population and prospectively examined the cortical signatures of susceptible HE patients with the non-invasive EEG recording method.

The estimated incidence of decompensated cirrhosis was about 34% in the newly diagnosed cirrhosis population, and more than half may suffer HE [19,20]. About 28.5% patients were diagnosed with secondary HE during hospitalization. The exclusion criteria of severe co-morbidity and trans-jugular intrahepatic portosystemic shunt cases may contribute to the relatively low rate of HE in this study [21]. In addition, the major etiology of cirrhosis was associated with hepatitis in more than half of the patients, and about 20% for alcohol-related cirrhosis in HE sub-group, which serves as a strong predictor of HE in cirrhosis patients [3].

Despite the etiologies, multiple risk factors were also considered to be related with the development of HE, including age, bilirubin, INR, creatinine, sodium, HE grading, presence of portal hypertension, minimal HE, and medications (PPIs, opiates, GABAergics, and benzodiazepines) [3,22,23]. This is consistent with our finding in this study that higher risks were associated with the usage of PPIs during hospitalization. However, the mechanism underlying the neurological dysfunction induced by PPIs remains very uncertain.

It is well known that the elevation of blood ammonia participates in the development of HE [24], of which the metabolism can be regulated by the intestinal microbiota directly or indirectly [25]. The application of PPIs can change the pH of digestive system, resulting in an unbalanced microenvironment for the intestinal microbiota [26,27]. Furthermore, the small intestinal bacterial overgrowth can also be induced directly by targeting the proton pump of the bacteria and fungi [26], which is associated with the bacterial translocation and HE development.

The early recognition of HE candidates in liver cirrhosis is essential for preventing HE and for improving clinical outcomes. However, identifying these patients at the early stages of neurological impairments remains challenging, such as minimal HE or covert HE, mainly due to the lack of clinically appreciable symptoms [28,29]. Several tools can be applied to assess the mild changes in metal conditions [30,31], which may be costly and hard to apply in clinical practices [29]. Alternatively, the non-invasive EEG recoding method has been routinely used in the diagnosis of neurological disorders, including epilepsy, autism spectrum disorders, and Alzheimer’s disease [10,11]. More recently, the application of its usage in the diagnosis of overt HE may provide us with an alternative and feasible approach for distinguishing the mild cases before potentially adverse interventions, such as PPI treatments in this study.

In the EEG experiment, eight cases (66%) were diagnosed with HE after one-week of PPI therapy, and they were all classified into minimal HE. The baseline EEG was performed at admission and we found significant decreasing spectral power densities of the alpha oscillations in the HE sub-group. Our preliminary evidence may reveal one possible link between cortical oscillatory activities and the HE’s development during PPI applications. Further studies with large sample sizes are needed to confirm the clinical efficiency of EEGs in the prediction of HE or minimal HE under PPI treatments. Despite PPI interventions, our data were consistent with previous findings that the disturbance of alpha oscillations is captured at the initial stage of HE [12,32].

It is well known that alpha waves play important roles in cognitive functions and dominate cortical activities during quiet wakefulness [33]. In addition, the reduction in alpha oscillations is associated with certain forms of neurological disorders, such as dementia [34]. In addition to the characteristic features of EEG patterns, the alpha oscillation may also serve as a target of neuromodulation for treating major depressive disorders and attention-deficit hyperactivity disorders [35,36,37]. Thus, we think that applying this index in future studies will be promising for examining its role in the identification of HE or minimal HE, or it can be a potential therapeutic target for mental dysfunctions in cirrhosis patients who take PPI treatments.

Currently, the potential mechanism underlying the abnormal rhythm of cortical oscillations caused by distinct drug usage remains unknown. For example, characteristic increasing beta frequencies were observed after the administration of benzodiazepines [38,39], which may induce EEG synchronizations and slow wave sleep to exert hypnogenic action at the level of the lower brain stem [40]. In contrast, alpha oscillatory activity has been modulated by multiple pharmacological agents via GABAergic, glutamatergic, cholinergic, and serotonergic receptors in the cortex and thalamus [41]. Recent evidence demonstrated that PPIs used against the core-cholinergic enzyme are responsible for biosynthesis of acetylcholine and contribute to the development of cognitive impairments [42]. Given the permeation kinetics via the blood–brain barrier [43], we assume that PPIs may regulate the concentration of acetylcholine and/or other neurotransmitters in the central nervous system, resulting in an altered phenotype with respect to alpha brain activities.

There were several limitations in this study. First, the main limitation of this study originates from the small sample size used in the EEG experiment, as well as the potential bias during patient selection. Thus, we may not conclude that the cortical patterns of alpha activities are generally caused by PPI treatments, but there may be one potential link between the brain function and medical therapy. Secondly, the application of PPIs may not be the only at-risk medication in cirrhosis; others such as benzodiazepines, opiates, and gamma aminobutyric acid may also contribute to the development of secondary HE. It is necessary to investigate the electrophysiological effects related with other commonly used drugs.

## 5. Conclusions

In this study, we evaluated the potential risk of HE development in cirrhosis patients. Multiple risk factors were independently associated with secondary HE in hospitalized patient with liver cirrhosis, including PPI usage, white blood cell and neutrophil counts, hemoglobin, prothrombin time activity, and blood urea nitrogen. Thus, our findings supported the susceptibility of secondary HE during PPI management. Furthermore, in the EEG experiment, we found one potential cortical mechanism underlying this neurological disease, is the suppression of alpha oscillations of the brain.

## Figures and Tables

**Table 1:** Classification of hepatic encephalopathy (HE) in patient with cirrhosis.

Revised HE Grading Criteria	Neuropsychiatric Symptoms	Nervous System Signs
Non-HE	Normal	Normal nervous system signs, normal neuropsychological test results
Minimal HE	Potential HE, no noticeable personality or behavioral changes	Normal nervous system signs, but abnormal neuropsychological test results
HE Grade One	Trivial and mild clinical signs, such as mild cognitive impairment, decreased attention, sleep disorders (insomnia and sleep inversion), euphoria, or depression	Asterixis can be elicited and neuropsychological tests are abnormal
HE Grade Two	Marked personality or behavioral changes, lethargy or apathy, slight orientation abnormality (time and orientation), decreased mathematical ability, dyskinesia, or unclear speech	Asterixis is easily elicited, and neurophysiological testing is unnecessary
HE Grade Three	Marked dysfunction (time and spatial orientation), abnormal behavior, semi-coma to coma, but responsive	Asterixis usually cannot be elicited. There is ankle clonus, increased muscle tone, and hyperreflexia. Neurophysiological testing is unnecessary
HE Grade Four	Coma (no response to speech and external stimuli)	Increased muscle tone or positive signs of the central nervous system. Neurophysiological testing is unnecessary

**Table 2:** Definition of recording site of the EEG.

ROI	Channel
Frontal site	FP_1_, FP_2_, F_3_, F_4_, F_7_, F_8_
Central site	C_3_, C_4_
Parietal site	P_3_, P_4_, P_7_, P_8_
Occipital site	O_1_, O_2_
Temporal site	T_7_, T_8_

ROI: Region of interest.

**Table 3:** Clinical manifestations and laboratory results at admission.

	HE (*n* = 74)	Non-HE (*n* = 186)	*p* Value
Sex, male, n (%)	59 (79.7)	130 (69.9)	0.071
Age (years)	56.65 ± 10.94	55.13 ± 12.58	0.583
WBC (×10^9^)	7.15 ± 4.82	5.04 ± 3.12	0.000 *
Hemoglobin (g/L)	103.93 ± 25.39	113.99 ± 22.64	0.002 *
Platelet (×10^9^)	91.76 ± 69.55	86.07 ± 57.25	0.678
Neutrophils (×10^9^)	5.31 ± 4.19	3.57 ± 2.83	0.000 *
ALT (U/L)	211.01 ± 532.61	210.55 ± 787.61	0.884
AST (U/L)	218.23 ± 456.52	242.06 ± 1138.16	0.439
TB (umol/L)	147.49 ± 145.90	100.74 ± 117.73	0.011 *
AKP (IU/L)	194.79 ± 170.60	181.28 ± 159.67	0.452
γ-GT (U/L)	111.73 ± 133.36	166.29 ± 280.14	0.557
Albumin (g/L)	26.31 ± 5.44	29.42 ± 6.18	0.000 *
BUN (mmol/L)	8.72 ± 5.44	6.26 ± 4.00	0.000 *
Creatinine(umol/L)	102.03 ± 62.74	91.22 ± 109.09	0.010 *
CRP (mg/L)	30.67 ± 43.54	20.84 ± 25.74	0.085
K^+^ (mmol/L)	3.97 ± 0.65	3.80 ± 0.54	0.029 *
Na^+^ (mmol/L)	136.21 ± 6.24	138.00 ± 3.83	0.080
PTA (%)	45.60 ± 17.14	57.85 ± 16.97	0.000 *
INR	1.80 ± 0.96	1.42 ± 0.32	0.000 *
Child, *n* (A/B/C)	0/16/58	21/113/52	0.000 *
MELD Scores	21.81 ± 7.91	16.47 ± 6.12	0.000 *
PPIs (%)	71 (95.95%)	142 (76.34%)	0.000 *
Pathogeny			0.507
Hepatitis B, *n* (%)	41 (55.41%)	111 (59.14%)	
Hepatitis C, *n* (%)	3 (4.05%)	10 (5.38%)	
Alcoholic liver, *n* (%)	15 (20.27%)	22 (11.83%)	
Hepatocellular carcinoma, *n* (%)	5 (6.76%)	18 (9.68%)	
others, *n* (%)	10 (13.51%)	25 (13.44%)	

WBC, white blood cell; ALT, glutamic pyruvic transaminase; AST, glutamic oxaloacetic transaminase; AKP, alkaline phosphatase; γ-GT, γ-glutamyl transpeptidase; BUN, blood urea nitrogen; CRP, C-reactive protein; TB, total bilirubin; PTA, prothrombin time activity; INR, international standard ratio; MELD, model for end stage liver disease; PPIs, proton pump inhibitors. * (*p* < 0.05) indicates that there is a statistical difference.

**Table 4:** Association between the development of HE and risk factors.

Covariates	Not Adjusted OR (95% CIs)	*p* Value	Adjusted OR (95% CIs)	*p* Value
WBC	1.155 (1.068–1.248)	<0.001	1.972 (1.299–2.993)	0.001
Neutrophils	1.161 (1.064–1.266)	0.001	0.505 (0.317–0.805)	0.004
Hemoglobin	0.982 (0.970–0.994)	0.003	0.978 (0.963–0.992)	0.003
PPIs	7.333 (2.201–24.438)	0.001	7.867 (2.166–28.575)	0.002
PTA	0.958 (0.941–0.975)	<0.001	0.955 (0.936–0.975)	<0.001
BUN	1.116 (1.052–1.185)	<0.001	1.104 (1.018–1.198)	0.017

WBC, white blood cell; PPIs, proton pump inhibitors; PTA, prothrombin time activity; BUN, blood urea nitrogen.

## Data Availability

The data presented in this study are available on request from the corresponding author. The data are not publicly available due to privacy and ethical reasons.
